# Color-Coded Compressive Spectral Imager Based on Focus Transformer Network

**DOI:** 10.3390/s25072006

**Published:** 2025-03-23

**Authors:** Jinshan Li, Xu Ma, Aanish Paruchuri, Abdullah Alrushud, Gonzalo R. Arce

**Affiliations:** 1 Key Laboratory of Photoelectronic Imaging Technology and System of Ministry of Education of China, School of Optics and Photonics, Beijing Institute of Technology, Beijing 100081, China; lijinshan0527@163.com; 2 Department of Electrical and Computer Engineering, University of Delaware, Newark, DE 19716, USA; aanishp@udel.edu (A.P.); alrushud@udel.edu (A.A.)

**Keywords:** hyperspectral imaging, compressive sensing, color-coded aperture, transformer

## Abstract

Compressive spectral imaging (CSI) methods aim to reconstruct a three-dimensional hyperspectral image (HSI) from a single or a few two-dimensional compressive measurements. Conventional CSIs use separate optical elements to independently modulate the light field in the spatial and spectral domains, thus increasing the system complexity. In addition, real applications of CSIs require advanced reconstruction algorithms. This paper proposes a low-cost color-coded compressive snapshot spectral imaging method to reduce the system complexity and improve the HSI reconstruction performance. The combination of a color-coded aperture and an RGB detector is exploited to achieve higher degrees of freedom in the spatio-spectral modulations, which also renders a low-cost miniaturization scheme to implement the system. In addition, a deep learning method named Focus-based Mask-guided Spectral-wise Transformer (F-MST) network is developed to further improve the reconstruction efficiency and accuracy of HSIs. The simulations and real experiments demonstrate that the proposed F-MST algorithm achieves superior image quality over commonly used iterative reconstruction algorithms and deep learning algorithms.

## 1. Introduction

Hyperspectral imaging technology has achieved success in the applications of target detection [1], food surveillance [2], environmental protection [3], biomedicine [4,5], and remote sensing [6]. A hyperspectral image (HSI) can be represented by a three-dimensional (3D) data cube containing two spatial dimensions (*x* and *y*) and one spectral dimension (*λ*). Traditional spectral imaging uses a two-dimensional (2D) detector to sample the 3D data cube by sequentially scanning the target scene along the spatial or spectral coordinates. However, this approach will prolong the data acquisition time, which is inadequate in some applications, especially for dynamic target imaging [7].

**Figure 1:**
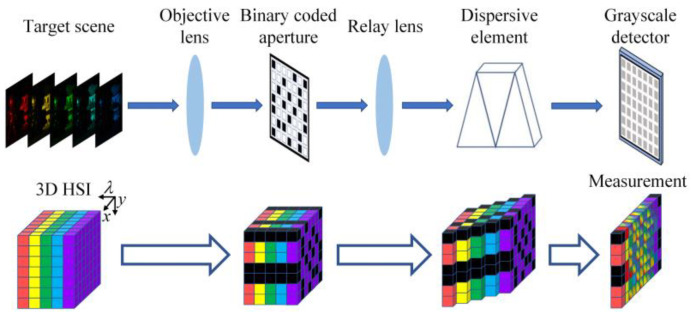
The schematic diagram of the traditional CSI system.

Compressive spectral imaging (CSI) relies on compressive sensing theory to solve the above problem [8,9]. The schematic diagram of a typical CSI system is shown in Figure 1 [10,11]. First, the HSI of the target scene is spatially modulated by a binary coded aperture, and then the spectral bands of the HSI are shifted by different displacements through a dispersive element. Afterwards, the encoded HSI is projected onto a grayscale detector along the spectral dimension to form the 2D compressive measurement. Finally, the 3D HSI can be reconstructed from a single or a few compressive measurements using the numerical algorithms.

However, traditional CSIs suffer from several limitations. Firstly, conventional CSI systems are composed of separate optical modulation elements, which prevent the system integration and miniaturization. Secondly, the encoding ability of the binary coded aperture is limited due to the lack of spectral modulation [12,13]; thus, multiple snapshots with varying coding patterns are necessary to further improve the reconstruction quality. However, switching the coding patterns will increase the cost and complexity of the spatial light modulator. Finally, most conventional HSI reconstruction algorithms for CSI systems are computationally intensive [14,15].

Recently, color-coded compressive spectral imaging (CCSI) was proposed for reducing the detection time [16,17]. The color-coded aperture (CCA) is used to replace the binary coded aperture and dispersive element in the traditional CSI system. The CCA is a 2D array composed of various optical filters with different spectral responses; thus, it has modulation capabilities in both the spatial and spectral domains. Previous studies proposed to attach the CCA to the camera sensor to obtain a compact system, and different fabrication methods for CCA were proposed. Specifically, Zhao et al. proposed a low-cost CCSI system based on the colored printed mask [18], which was fabricated by a consumer-level printer with color inks. However, the uncontrollable placement of ink droplets may lead to some deviations from the designed coding pattern. A spectral imaging system based on a diffractive optical element and a CCA was proposed in [19]. This CCA was produced using an ordinary film photography technique, which improved manufacturing accuracy at low cost. However, the periodic arrangement of the CCA limited its modulation freedom. In addition, the method using a diffractive lens in concert with a CCA provides limited field of view. Yako et al. proposed a hyperspectral camera based on Fabry–Pérot filters [20], where multiple filters with different spectral responses were randomly arranged to obtain color coding. This kind of CCA required advanced manufacturing techniques that increased the fabrication cost. Zhang et al. developed a low-cost multi-spectral camera at tens-of-megapixel spatial resolution [21]. In that work, the color-coded mask was generated by imaging a binary coding mask through a disperser, but the wavelength-dependent dispersive effect limited the modulation freedom. In general, traditional CCAs face a trade-off among coding freedom, modulation accuracy, and fabrication cost. It is preferrable to design a low-cost freeform CCA component that can be fabricated precisely.

In addition, the CCSI can reconstruct the HSI using only one snapshot without switching the coding pattern, thus saving the detection time. However, the compression ratio of the original HSI data over the compressive measurement is very high, which brings in a big challenge for the reconstruction algorithms. To address this problem, deep learning technology was introduced to improve the reconstruction efficiency and accuracy of the CCSI. In the past, convolutional neural networks (CNNs) were used to construct the end-to-end mapping models between the compressive measurement and the reconstructed HSI [18,19,20]. Transformer-based networks were also proposed to further improve the reconstruction quality of traditional CSI and CCSI by capturing the long-range inter-spectra dependencies of the target HSIs [21,22,23]. However, the convolution-based down-sampling and up-sampling operations used in the Transformer-based models may lose information of feature maps, which was detrimental to the reconstruction accuracy. Moreover, there is currently a lack of training datasets for real CCSI systems, which induces difficulty for the deep learning methods to reconstruct high-quality HSI in real applications.

**Figure 2:**
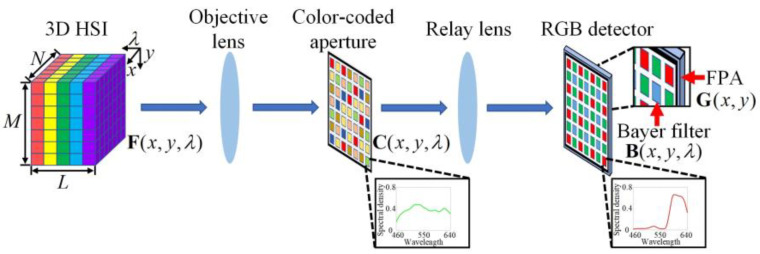
Schematic diagram of the LCCSI system, where the HSI data cube is firstly projected through the objective lens on the CCA and then projected on the RGB detector by the relay lens.

This paper proposes a low-cost color-coded compressive spectral imager (LCCSI), and a corresponding deep learning approach, dubbed Focus-based Mask-guided Spectral-wise Transformer (F-MST), that can obtain high-quality reconstruction for the LCCSI system. Figure 2 shows the schematic diagram of the LCCSI system. It jointly uses a CCA fabricated using a color film and an RGB detector to achieve higher degrees of freedom in the spatio-spectral modulations. First, the target’s light field is projected through an objective lens on the CCA. The CCA is produced by imaging a color-coded pattern onto a transparent color film using conventional film photography, which offers varying spectral modulations on different pixels. The HSI data cube is modulated by the CCA on both spatial and spectral domains and then projected on an RGB detector by a relay lens. The HSI data cube is modulated again by the Bayer filter integrated in the RGB detector. The Bayer filter is a three-color filter array, i.e., red, green, and blue filters. Finally, the twice-encoded HSI is captured by the focal plane array (FPA) of the detector to form a 2D compressive measurement. The joint spatial and spectral modulations of the CCA and Bayer filter can further increase the modulation freedom, thus obtaining an incoherent sensing matrix for the LCCSI system, which is beneficial to improve the HSI reconstruction quality. The proposed CCA is very thin in volume; thus, it can be tightly attached on the detector surface to miniaturize the LCCSI system.

In order to reconstruct the spectral data cube, this work also develops the F-MST network that is inspired by the Mask-guided Spectral-wise Transformer (MST) network designed for the traditional CSI system [22]. We embed the focus-based down-sampling and up-sampling modules in the MST network to improve the reconstruction accuracy. To overcome the problem of lacking training sets, we first use the simulation data to pre-train the deep learning models, and then we retrain them with the real dataset collected by the LCCSI testbed established by our group. The proposed LCCSI system and F-MST reconstruction network are verified and assessed based on both simulations and real experiments. These show that the proposed F-MST method achieves superior reconstruction performance over the commonly used iterative reconstruction algorithms (GPSR [24], TwIST [25], and GAP-TV [26]) and some other state-of-the-art learning-based algorithms (TSA-Net [27] and MST [22]).

The main contributions of this paper are summarized as follows. This work proposes a low-cost LCCSI system based on a cascaded encoding method and a corresponding supervised learning reconstruction algorithm for compressive spectral imaging. The combination of the film-based CCA and the Bayer filter array of the RGB detector can effectively enhance the coding freedom of the entire optical system. The proposed F-MST algorithm utilizes the focus-based down-sampling and up-sampling modules to maintain more feature information and improve reconstruction quality. The proposed LCCSI system and F-MST algorithm have the potential to be used in the miniaturized and high-quality hyperspectral imaging technology.

## 2. Imaging Model of LCCSI System

The imaging process of the LCCSI system is shown in Figure 2. The HSI of the target scene is represented as $F\in {\mathbb{R}}^{M\times N\times L}$ with the spatial size of M×N and the spectral depth of L. The coding effects of the CCA and the Bayer filter are represented by the matrices of $C\in {\mathbb{R}}^{M\times N\times L}$ and $B\in {\mathbb{R}}^{M\times N\times L}$, respectively. The CCA consists of a 2D array of color pixels with random arrangement, where each color pixel has a pre-defined spectral response that can transmit specific components of the light spectrum and suppress other components. The Bayer filter is a 2D array arranged in a 2×2 cycle consisting of three kinds of optical filters (red, green, and blue). It is noted that C and B have the same dimensionality. These two matrices are composed of the spectral modulation curves (along the dimension L) over all of the spatial coordinates (along the dimensions M×N). The voxels of C and B are defined as C(x,y,λ)∈[0,1] and B(x,y,λ)∈[0,1], respectively. Finally, the 2D compressive measurement on the detector is denoted by $G\in {\mathbb{R}}^{M\times N}$. Then, the imaging model of the LCCSI system is given by

(1) $$G(x,y)=\sum_{\lambda }^{L}T(x,y,\lambda )\cdot F(x,y,\lambda ),$$

where $T\in {\mathbb{R}}^{M\times N\times L}$ is referred to as the cascade coding cube that represents the total coding effects attributed to both the CCA and Bayer filter. It can be formulated as

(2) T(x,y,λ)=C(x,y,λ)⋅B(x,y,λ).

Next, we transform Equation (1) to a matrix multiplication format. Let $g\in {\mathbb{R}}^{MN\times 1}$ and $f\in {\mathbb{R}}^{MNL\times 1}$ be the vectorized representations of G and F, respectively. Then, Equation (1) is rewritten as

**Figure 3:**
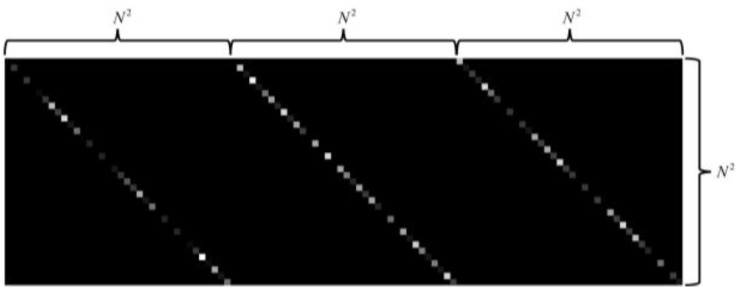
Illustration of sensing matrix for LCCSI system with M=N=6 and L=3.

(3) g=Hf+ω,

where $H\in {\mathbb{R}}^{MN\times MNL}$ represents the sensing matrix of the LCCSI system, and $\omega \in {\mathbb{R}}^{MN\times 1}$ represents the measurement noise. The sensing matrix H is the 2D representation of the cascade coding cube T. Figure 3 provides an intuitive illustration of the sensing matrix for one snapshot with the spatial dimensions M=N=6 and the spectral dimension L=3. The three groups of diagonal elements in Figure 3 correspond to the modulation coefficients in the three spectral bands (L=3), where the grayscale elements from black to white represent the element values from 0 to 1. It is shown that the sensing matrix is not binary but contains lots of grayscale elements with different values. This comes from the freeform color coding and brings in more freedom in the spatio-spectral modulations.

Reconstructing the 3D HSI data cube from the 2D measurement is an underdetermined problem that can be solved by compressive sensing methods. According to the highly correlated property of HSI along the spatial and spectral dimensions, f can be sparsely represented as f=Ψθ, where Ψ and θ denote the sparse basis matrix and sparse coefficient vector, respectively. In this paper, the basis matrix is defined as $\Psi =\Psi_{1}⊗\Psi_{2}$, where ⊗ denotes the Kronecker product, $\Psi_{1}$ is the 2D wavelet Symmlet 8 basis in the spatial domain, and $\Psi_{2}$ is the one-dimensional discrete cosine basis in the spectral domain. The wavelet Symmlet 8 basis can effectively capture the components of different frequencies at multiple scales in the spatial dimension, while the discrete cosine basis can maintain the major low-frequency characteristics of the spectrum. The HSI of the target scene can be reconstructed by solving the following optimization problem:

(4) $$\tilde{\theta }=\arg\underset{\theta }{\min}\left\{{\left\|g-H\Psi \theta \right\|}_{2}^{2}+\mu {\left\|\theta \right\|}_{1}\right\},$$

where $||\cdot |{|}_{2}$ and $||\cdot |{|}_{1}$ denote the $l_{2}$-norm and $l_{1}$-norm respectively, and μ is the regularization parameter. As mentioned in Section 1, deep learning approaches have been recently introduced to reconstruct the HSI directly from the measurement rather than iteratively updating the solutions by gradient-based algorithms. Our proposed deep learning method will be described in Section 4.

## 3. Experimental System of LCCSI

**Figure 4:**
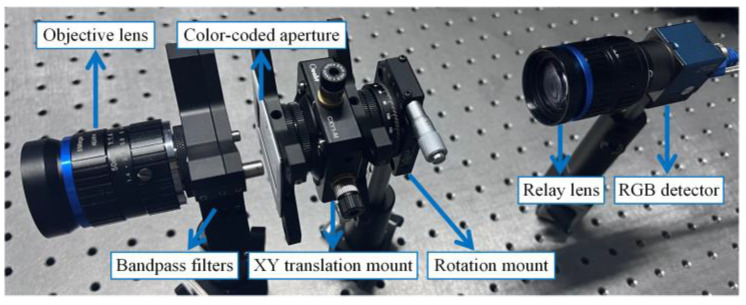
The experimental system of LCCSI established by our group.

In order to verify the proposed LCCSI method, our group established an experimental system of the LCCSI as shown in Figure 4. This system consists of an objective lens (FM5014-8MP, CW Lens, Shenzhen, China), a pair of bandpass filters (GCC-300115 and GCC-211002, Daheng Optics, Beijing, China), a CCA, an XY translation mount (CXY1-M, OEABT, Guangzhou, China), a precision rotation mount (S/N0007, OEABT, Guangzhou, China), a relay lens (FM3514-10MP-A, CW Lens, Shenzhen, China), and an RGB detector (MER2-231-41U3C, Daheng Imaging, Beijing, China). The focal lengths of the objective lens and relay lens are 50mm and 35 mm, respectively. The bandpass filters are placed behind the objective lens to limit the spectrum range of HSI from 450 nm to 650 nm. The XY translation mount and rotation mount are used to finely adjust the position and rotation angle of the CCA, thus ensuring the precise pixel match between the CCA and the RGB detector.

**Figure 5:**
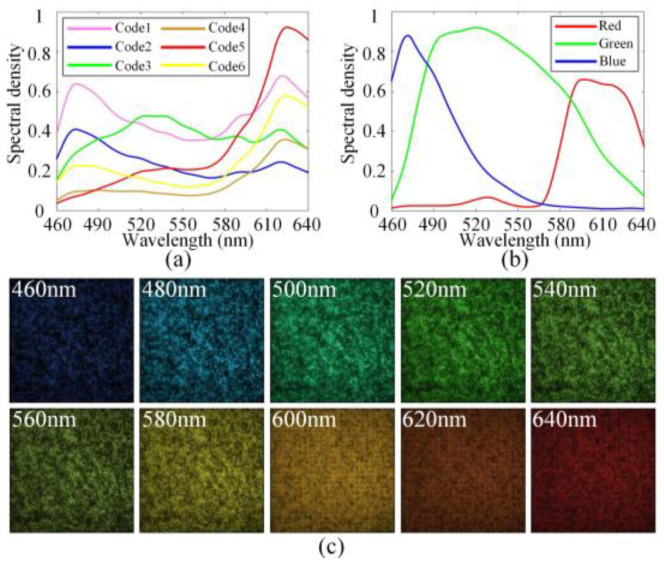
(**a**) Spectral modulation curves of the six codes in CCA; (**b**) the spectral modulation curves of the red, green, and blue filters contained in Bayer filter; (**c**) images of the calibrated coding cube $T^{\prime }$ for ten selected spectral bands.

The CCA in our system is produced by the research group at University of Delaware using conventional film photography, and its production process is similar to that of the CCA in [19]. Compared to the CCA with periodic arrangement in [19], our CCA adopts a completely random arrangement, which has higher modulation freedom. It was shown in [28] that the CCA with more than five color codes could achieve good reconstruction results. Thus, we use six color codes in our CCA. We use the spectrometer (USB2000+, Ocean Optics, Orlando, FL, USA) to obtain the spectral curves of a variety of color codes, and then we select six distinct codes that cover the visible spectrum to form the coding pattern with a random arrangement. Figure 5a shows the spectral modulation curves of the six codes in the CCA.

In our previous work, we designed a compact compressive spectral imaging system, where the film-based coding mask was attempted to be attached to the surface of the detector [29]. However, that system lacks the relay imaging scheme; thus, it is hard to achieve the perfect matching between the pixels on the CCA and the detector. Some advanced manufacturing methods may realize the high-resolution CCA with precise pixel size control, but this will significantly increase the manufacturing difficulty and cost of the CCA. Additionally, there is a protective glass on the detector’s sensor, which creates a gap between the CCA and the detector. This covering glass and air gap will induce refraction and diffraction effects, which may cause crosstalk among adjacent encoded pixels, causing the actual encoding process to deviate from the ideal encoding model.

To address these issues, this paper proposes an LCCSI system based on a relay imaging scheme as shown in Figure 4, where the CCA pixel size does not need to be consistent with the detector pixel size, thereby reducing the manufacturing difficulty of the CCA. Furthermore, we introduce a matching scheme between one CCA pixel and a 2×2 Bayer filter array, which not only relaxes the requirement on the resolution of the CCA but also enhances the system’s modulation freedom. The CCA used in this work contains a 128×128 color-coded array with a pixel size of 62.5 μm. This array is combined with a 256×256 Bayer filter array of the RGB detector to double the spatial resolution of the cascade coding cube T, which means that one CCA pixel is matched with a 2×2 cycle array of the Bayer filter. Through this specific matching mechanism, the modulation freedom of the coding cube is increased in both the spatial and spectral domains. In the spatial dimension, this matching procedure doubles the spatial size of T from 128×128 to 256×256, which enhances the spatial modulation freedom of the coding cube. In the spectral dimension, the red, green, and blue filters contained in the 2×2 Bayer cycle array have different spectral modulation curves, as shown in Figure 5b. When a CCA pixel is matched with a 2×2 Bayer filter cycle, more diverse spectral modulation curves can be generated, thereby enhancing the spectral modulation freedom of the coding cube. It is notable that a 2×2 Bayer filter cycle of the RGB camera contains two green filters with the same spectral response. This slightly reduces the diversity of spectral modulation in the coding cube. However, considering the cost and generalization, we use a common RGB camera as the detector for our LCCSI system.

However, the modulation behavior of the real experimental system may deviate from the designed target due to the non-ideal fabrication conditions of the CCA and the system assembly errors. In addition, the strict pixel matching between the CCA and RGB sensor is difficult to achieve through manual adjustment. Those problems may introduce errors in the imaging model, thus degrading the reconstructed result of the LCCSI system. To solve the above problems, after manually matching the pixels between the CCA and RGB sensor to the utmost, we need to calibrate the coding cube T in Equation (1) for the real LCCSI system, and the calibrated coding cube is denoted by $T^{\prime }$. It is noted that $T^{\prime }$ conforms to the real modulation process of the LCCSI system. In particular, we illuminate the CCA with a monochromatic light source that is composed of a xenon lamp light source (GLORIA-X500A, ZOLIX, Beijing, China) and a monochromator (Omni-λ300i, ZOLIX, Beijing, China), scanning over nineteen spectral bands with the center wavelengths from 460 nm to 640 nm. The xenon lamp light source first provides composite light illumination covering the entire visible spectrum for the monochromator. Then, the monochromator uses an internal grating to emit monochromatic light, which is transmitted through an optical fiber to an annular illuminator, ultimately achieving monochromatic light illumination for the LCCSI system. For each spectral band, we record the image of the CCA using the RGB detector. Those images are exactly the 2D transmittance matrices of the calibrated coding cube $T^{\prime }$ in the corresponding spectral bands. Then, the $T^{\prime }$ is obtained by stacking all transmittance matrices together along the spectral dimension. Figure 5c shows the images of the $T^{\prime }$ for ten selected spectral bands, where the spatial size of the coding cube is 256×256.

## 4. HSI Reconstruction Based on F-MST Network

A Transformer-based F-MST network was developed to achieve fast and high-quality reconstruction of HSIs. The proposed F-MST network is developed from the MST network applicable to the traditional CSI system [22]. Different from the original MST network, the F-MST network removes the shift modules [22] corresponding to the dispersive effect in the traditional CSI system. Then, the MASK [22] corresponding to the binary coded aperture of traditional CSI is replaced by the calibrated coding cube $T^{\prime }$ of the proposed LCCSI system. Besides, the convolution-based down-sampling and up-sampling operations [22] in the MST network are replaced by the focus-based down-sampling and up-sampling modules to improve the reconstruction accuracy.

**Figure 6:**
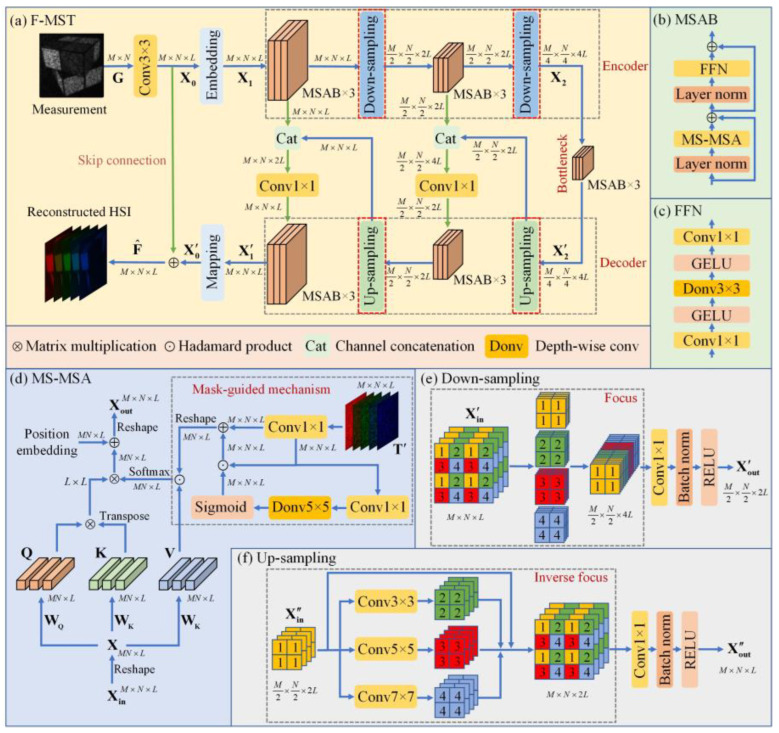
Schematic diagram of the proposed F-MST network: (**a**) the overall structure of F-MST network; (**b**) the components of MSAB; (**c**) the components of FFN; (**d**) the structure of MS-MSA; (**e**) the structure of focus-based down-sampling module; (**f**) the structure of focus-based up-sampling module.

Figure 6a shows the overall structure of the proposed F-MST network, which is composed of a 3×3 convolutional layer, an embedding layer, an encoder, a bottleneck, a decoder, and a mapping layer. On the whole, this network inherits the basic framework of the U-net [30]. First, the compressive measurement G of the LCCSI system is imported into the 3×3 convolutional layer, which expands the spectral dimension and generates the feature map $X_{0}\in {\mathbb{R}}^{M\times N\times L}$. Then, the embedding layer (3×3 convolutional layer) is used to map $X_{0}$ into another feature $X_{1}\in {\mathbb{R}}^{M\times N\times L}$. Afterwards, $X_{1}$ is inputted into the encoder, which contains two sets of three Mask-guided Spectral-wise Attention Blocks (MSABs) [22] and two down-sampling modules. The encoder outputs the feature map $X_{2}\in {\mathbb{R}}^{\frac{M}{4}\times \frac{N}{4}\times 4L}$, which then passes through the bottleneck containing three MSABs. The bottleneck outputs the feature map $X_{2}^{\prime }\in {\mathbb{R}}^{\frac{M}{4}\times \frac{N}{4}\times 4L}$, which serves as the input of the following decoder. The decoder contains two sets of three MSABs and two up-sampling modules. As shown in Figure 6a, the skip connections indicated by the green arrows are used for feature fusion between the encoder and decoder, where the channel concatenation and the 1×1 convolutional layer are used to reduce the information loss caused by the down-sampling operations. The shallow and deep features are fused through skip connections to improve the reconstruction quality of HSIs. The decoder outputs the feature map $X_{1}^{\prime }\in {\mathbb{R}}^{M\times N\times L}$, which is then converted to the feature map $X_{0}^{\prime }\in {\mathbb{R}}^{M\times N\times L}$ through the mapping layer (3×3 convolutional layer). Finally, the feature map $X_{0}^{\prime }$ is added to the feature map $X_{0}$ to obtain the reconstructed HSI $\hat{F}$.

Figure 6b shows the structure of the MSAB, which consists of a Feed-Forward Network (FFN), a Mask-guided Spectral-wise Multi-head Self-Attention (MS-MSA) module, and two layer normalization functions [22]. Figure 6c,d illustrate the structures of the FFN and the MS-MSA module, respectively. The MS-MSA can capture the long-range inter-spectra dependencies, which is conducive to learn the mapping functions between the 2D measurement and the 3D HSI [22]. In addition, the mask-guided mechanism (MM) [22] based on the proposed calibrated coding cube $T^{\prime }$ is used to guide the network to focus on the regions with highly credible information in both the spatial and spectral dimensions. This mechanism can further improve the reconstruction quality.

Next, we describe the differences between the proposed F-MST network and the original MST network in [22]:

(1) We remove the shifting modules [22] in the MST network corresponding to the dispersive component of the traditional CSI system. Then, in order to adapt the network to the LCCSI system, we add a convolutional layer before the embedding layer to expand the spectral dimensionality of compressive measurement G to L.

(2) The mask [22] in the MM module of the MST network corresponds to the binary coded aperture in the traditional CSI system. So, it is replaced by the 3D calibrated coding cube $T^{\prime }$ of the proposed system to enhance the attention to the regions with highly credible information in both the spatial and spectral domains.

(3) The convolution-based down-sampling and up-sampling operations [22] in the MST network are replaced by the focus-based down-sampling and up-sampling modules, which can reduce the loss of feature map information and thus improve the reconstruction accuracy of HSIs. The structure of the focus-based down-sampling module is shown in Figure 6e. The focus module firstly obtains four feature maps of half spatial size by interval sampling and then concatenates them along the channel dimension. Combined with a 1×1 convolutional layer, a batch normalization function, and a ReLU activation function, the focus-based down-sampling module can convert the input feature map $X_{in}^{\prime }\in {\mathbb{R}}^{M\times N\times L}$ to the output feature map $X_{out}^{\prime }\in {\mathbb{R}}^{\frac{M}{2}\times \frac{N}{2}\times 2L}$. On the other hand, the structure of the focus-based up-sampling module is shown in Figure 6f. The inverse focus module uses convolutions with different kernel sizes to obtain three new feature maps, which together with the original feature map can achieve up-sampling in the spatial domain. Combined with a 1×1 convolutional layer, a batch normalization, and a ReLU function, the focus-based up-sampling module can convert the input feature map $X_{in}^{\prime \prime }\in {\mathbb{R}}^{\frac{M}{2}\times \frac{N}{2}\times 2L}$ to the output feature map $X_{out}^{\prime \prime }\in {\mathbb{R}}^{M\times N\times L}$. The focus module can retain all the information of the original feature map in the down-sampling process. In addition, the convolutions with different kernel sizes can capture the feature information at different scales in the up-sampling process. With the help of the focus-based down-sampling and up-sampling modules, the proposed F-MST network can further improve the reconstruction quality of HSIs.

## 5. Simulation and Experimental Results

### 5.1. Experimental Settings

**Figure 7:**
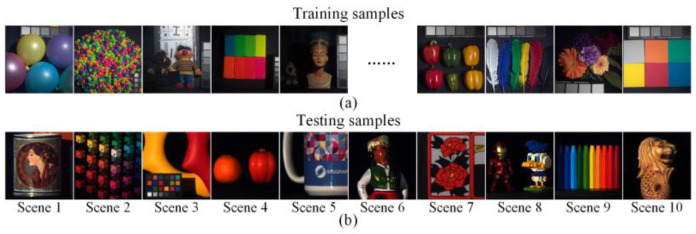
The RGB images of the public simulation datasets. The RGB images of (**a**) the partial training samples, and (**b**) the ten testing samples.

**Simulation data**: Firstly, we verify the proposed LCCSI system and F-MST reconstruction algorithm on the simulation data. We use the public datasets CAVE [31] and KAIST [32] to train the deep neural networks. The CAVE dataset includes thirty-two HSIs with the spatial size of 512×512, and the KAIST dataset includes thirty HSIs with the spatial size of 2704×3376. The HSIs in both datasets include thirty-one spectral bands with center wavelengths from 400 nm to 700 nm. Following the settings of the MST in [22], we use the CAVE dataset for training and ten scenes of the KAIST dataset for testing. Due to the different collection environments and conditions between the training and testing datasets, the reconstruction results can demonstrate the generalization ability of the trained model. By implementing random flipping and rotation operations for data augmentation, the number of HSIs in the simulation training dataset is increased from 32 to 205, greatly reducing the risk of bias and overfitting in the simulation. To match the dimensionality of the calibrated coding cube $T^{\prime }$ for the real LCCSI system, we select nineteen spectral bands for the simulation HSIs with center wavelengths from 460 nm to 640 nm. The training and testing datasets need to possess the same spatial resolution and the same number of spectral bands before being fed into the model. Therefore, the training samples are randomly cropped into HSIs with a spatial size of 256×256 in each training epoch. The ten testing samples are also cropped into HSIs with a spatial size of 256×256. Figure 7a and Figure 7b show the RGB images corresponding to the training samples and ten testing samples used for simulations, respectively.

**Figure 8:**
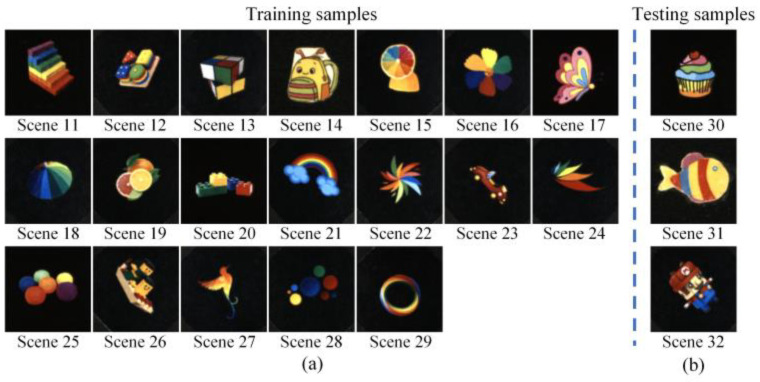
The RGB images of the twenty-two real scenes for experiments. The RGB images of (**a**) the nineteen real scenes used for training, and (**b**) the three real samples used for testing.

**Real data**: In this work, we also verify the proposed methods on the real testbed established by our group. The details of the experimental testbed were described in Section 3. Compared to the ideal simulation scenes, the system noise in the real scenes may reduce reconstruction performance when applying the simulation model to the experimental data. Therefore, we established a real dataset containing twenty-two real scenes to train the model in the experiments. We first collect the HSIs of twenty-two real scenes. The ground-truth HSIs are captured by scanning those scenes using a monochromatic light source. Then, the compressive measurements of those scenes are captured using an LED light source (GCI-060411, Daheng Optics, Beijing, China). All of the real HSIs have the spatial size of 256×256 and include nineteen spectral bands with center wavelengths from 460 nm to 640 nm. Figure 8 shows the RGB images of the twenty-two real scenes, where nineteen scenes are used for training and the other three scenes are used for testing.

**Implementation details**: In this work, we compare the proposed F-MST network with some other popular reconstruction algorithms, including some traditional numerical algorithms such as the GPSR [24], TwIST [25], and GAP-TV [26] algorithms, as well as some deep learning approaches such as the TSA-Net [27] and MST network [22]. For the deep learning approaches, the deep neural networks are trained on the NVIDIA GeForce RTX 2080 Ti GPU. The loss functions are the root mean square error (RMSE) between the reconstructed and ground-truth HSIs. The number of training epochs is 300, and the batch size is five. The learning rate is 0.0004, which decreases by half for every fifty training epochs. In this paper, four kinds of metrics are used to assess the reconstruction accuracy of HSIs. The first one is the peak signal-to-noise ratio (PSNR) of the reconstructed images, the second one is the structural similarity (SSIM) of the reconstructed images, and the third one is the correlation between the reconstructed spectral curves and the ground truths. The fourth one is the spectral angle mapper (SAM) between the reconstructed spectral curves and the ground truths. In addition, the efficiencies of different algorithms are compared based on the average runtimes.

### 5.2. Simulation Results

In this section, we reconstruct the HSIs from a single compressive measurement on the simulation dataset. Table 1 shows the reconstruction PSNRs, SSIMs, and the average runtimes over the ten scenes based on the six algorithms mentioned above, where the best indicator values are represented in bold. It shows that the proposed F-MST network outperforms the GPSR, TwIST, GAP-TV, TSA-Net, and MST methods by 10.06 dB, 8.80 dB, 10.14 dB, 0.86 dB, and 0.33 dB in the average PSNR over the ten scenes, respectively. In addition, in the average SSIM over the ten scenes, the F-MST network is 0.001 lower than the MST network, but it outperforms the GPSR, TwIST, GAP-TV, and TSA-Net methods by 0.176, 0.109, 0.115, and 0.005, respectively. Furthermore, the average runtimes of the three deep learning approaches are much shorter than the other three iterative algorithms, demonstrating the superiority of deep learning methods in terms of computational efficiency.

**Figure 9:**
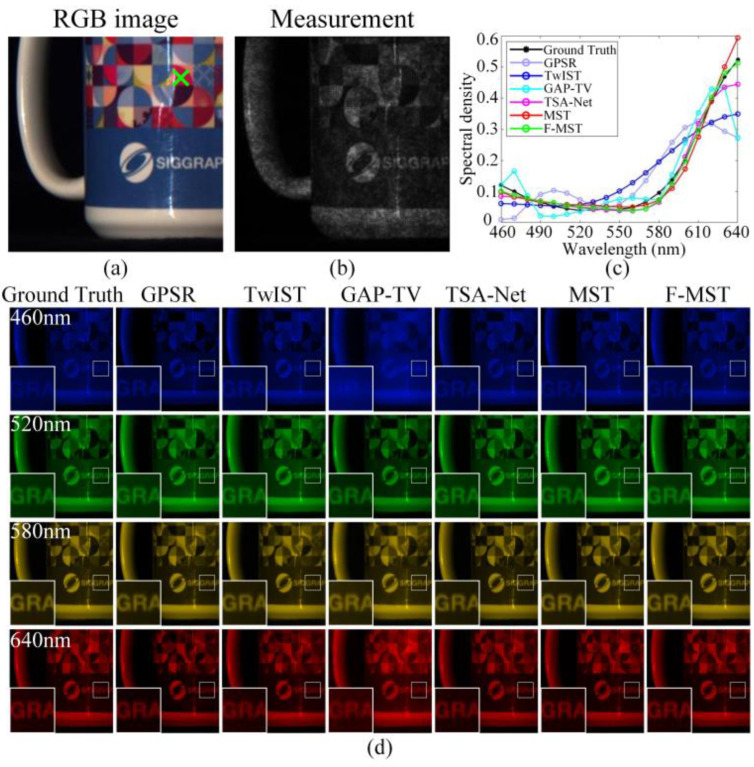
Original and reconstructed results of Scene 5 obtained by different algorithms using simulation data: (**a**) the RGB image; (**b**) the compressive measurement; (**c**) the ground-truth and reconstructed spectral curves corresponding to the green cross in the RGB image; (**d**) the ground-truth and reconstructed HSIs within four selected spectral bands.

Figure 9a shows the RGB image of Scene 5 given in Figure 7, and Figure 9b shows the corresponding compressive measurement. Figure 9c shows the original and reconstructed spectral curves obtained by different algorithms corresponding to the spatial point marked by the green cross in Figure 9a. In addition, the correlations and SAMs between the ground-truth and reconstructed spectral curves are displayed in the last two rows of Table 1. They show that the proposed F-MST method achieves the highest reconstruction accuracy in the spectral dimension among all of the six algorithms. Figure 9d shows the ground-truth and reconstructed HSIs of Scene 5 within four selected spectral bands, and the small image regions within the white blocks are magnified and shown in the bottom left corners. We can observe that the deep learning algorithms can reconstruct clearer images compared to the traditional iterative algorithms. In addition, the simulation reconstruction results of Scene 8 are also shown in Figure S1 of the Supplementary Materials. According to the simulation results, although the F-MST algorithm is slightly inferior to the MST algorithm in terms of SSIM and reconstruction efficiency, considering all of the evaluation metrics for the ten scenes, the F-MST still outperforms other algorithms in comprehensive performance.

### 5.3. Experimental Results

This section verifies the proposed methods using experimental data. In order to prevent the bias and overfitting problems due to the lack of sufficient training data, we first use the simulation data to pre-train the three deep learning models, and then we retrain them with the real dataset collected by our LCCSI system. In addition, it should be emphasized that the real coding cube $T^{\prime }$ calibrated by the LCCSI system is used in the simulation pre-training stage, which takes into account the system assembly errors and conforms to the real experimental conditions. The aforementioned training process will effectively alleviate the bias and overfitting issues to some extent. Table 2 shows the reconstruction PSNRs, SSIMs, and average runtimes of different methods based on the three real testing scenes in Figure 8. In this table, the best indicator values are represented in bold. It shows that the F-MST network outperforms the GPSR, TwIST, GAP-TV, TSA-Net, and MST algorithms by 8.77 dB, 7.24 dB, 9.87 dB, 1.96 dB, and 0.97 dB in the average PSNR, respectively. In addition, the F-MST method achieves higher reconstruction SSIMs on average compared to other algorithms. In addition, the average runtimes of the six algorithms are close to those presented in Table 1.

**Figure 10:**
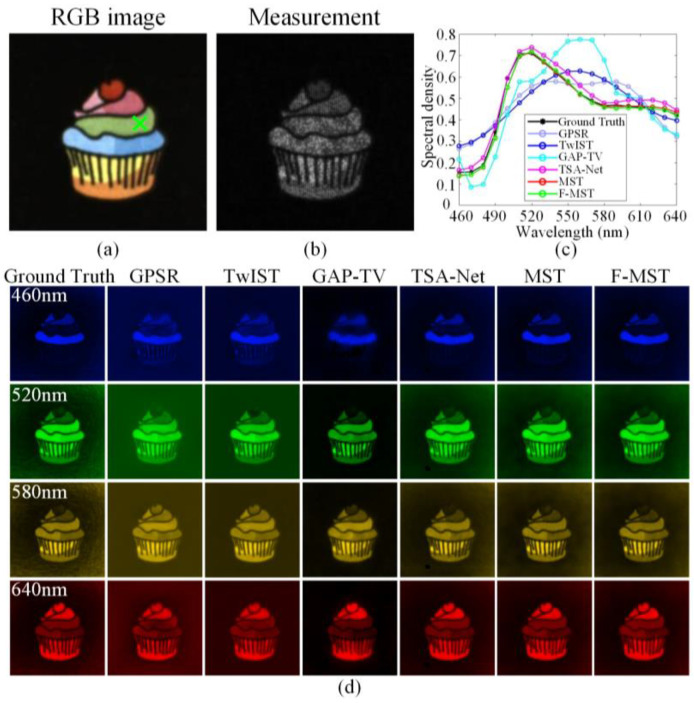
Original and reconstructed results of Scene 30 obtained by different algorithms using real experimental data: (**a**) the RGB image; (**b**) the compressive measurement; (**c**) the ground-truth and reconstructed spectral curves corresponding to the green cross in the RGB image; (**d**) the ground-truth and reconstructed HSIs within four selected spectral bands.

Figure 10a,b show the RGB image and compressive measurement of Scene 30 given in Figure 8, respectively. Figure 10c shows the ground-truth and reconstructed spectral curves corresponding to the spatial point marked by the green cross in Figure 10a. In addition, the last two rows of Table 2 display the correlations and SAMs between the aforementioned ground-truth and reconstructed spectral curves. Figure 10d shows the ground-truth and reconstructed HSIs of Scene 30 within four selected spectral bands. In addition, the experimental reconstruction results of Scene 32 are also shown in Figure S2 of the Supplementary Materials. The real experiments also prove the superiority of the proposed F-MST algorithm compared to the other five algorithms.

### 5.4. Ablation Study

As mentioned above, the proposed LCCSI system uses a cascade coding scheme involving both the CCA and Bayer filter to increase the modulation freedom. In order to prove the effectiveness of the cascade coding scheme, we conduct an ablation experiment using the LCCSI system without the CCA, which only uses the Bayer filter to modulate the light field and then reconstructs the HSIs. We call the LCCSI system without the CCA the “Only Bayer system”.

**Figure 11:**
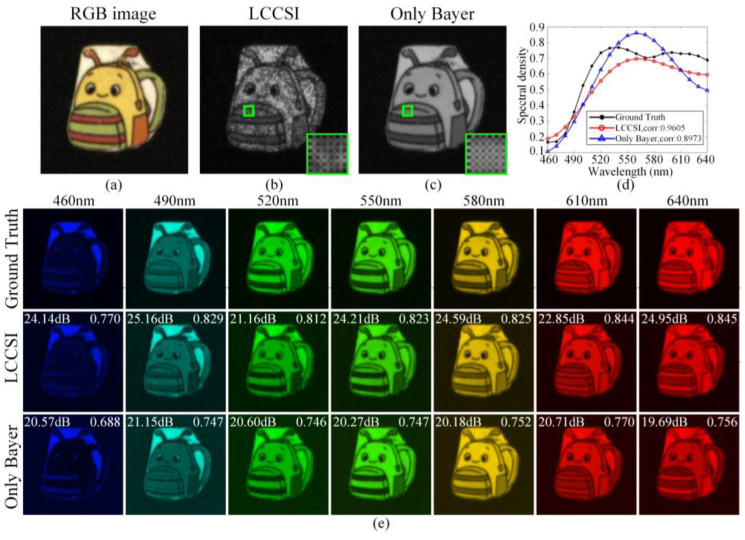
Original and reconstruction results of Scene 14 obtained by the LCCSI system and the Only Bayer system: (**a**) the RGB image; (**b**) the measurement captured by LCCSI system; (**c**) the measurement captured by Only Bayer system; (**d**) the spectral curves corresponding to the spatial region within the green boxes on the measurements; (**e**) the ground-truth and reconstructed HSIs within seven selected spectral bands.

Figure 11 compares the reconstructed results of Scene 14 in Figure 8 using the TwIST algorithm. Figure 11a shows the RGB image of Scene 14. Figure 11b and Figure 11c respectively illustrate the compressive measurements captured by the proposed LCCSI system and the Only Bayer system, where the small regions in the green boxes are magnified and shown in the bottom right corners. Figure 11d shows the ground-truth and reconstructed spectral curves corresponding to the spatial regions marked by the green boxes on the measurements. Compared with the Only Bayer system, the spectral correlation obtained by the LCCSI system is increased by 0.0632.

Figure 11e shows the ground-truth and reconstructed HSIs of Scene 14 within seven selected spectral bands, where the reconstruction PSNRs and SSIMs are respectively presented in the upper left corners and upper right corners in each image. For the LCCSI system, the average reconstruction PSNR over all spectral bands is 23.63 dB, and the average reconstruction SSIM is 0.823. For the Only Bayer system, the average reconstruction PSNR is 20.53 dB, and the average reconstruction SSIM is 0.747. The ablation experiments demonstrate the advantage of the proposed cascade coding scheme in the LCCSI system in terms of reconstruction performance.

## 6. Conclusions

This paper proposed the low-cost LCCSI system and the corresponding F-MST deep neural network to reduce the complexity of the CSI system and to improve the accuracy and efficiency of HSI reconstruction. The proposed method jointly modulated the spatial and spectral light field by using the CCA in tandem with the RGB detector to improve the modulation capacity in both spatial and spectral domains. In addition, the proposed CCA can be easily fabricated using the ordinary photography technique, which offered higher degrees of modulation freedom. Moreover, the proposed F-MST network used the focus-based down-sampling and up-sampling modules to effectively improve the reconstruction performance of the LCCSI system. The superiority of the proposed method was demonstrated on both simulation and experimental datasets. In addition, the ablation study was conducted to prove the benefit of the cascade coding scheme used in the LCCSI system. In the future, the methods to further miniaturize the LCCSI system will be studied.

## Figures and Tables

**Table 1:** Reconstruction PSNRs (dB), SSIMs, average runtimes, correlations, and SAMs of different methods based on ten selected scenes using simulation data.

Methods	GPSR PSNR/SSIM	TwIST PSNR/SSIM	GAP-TV PSNR/SSIM	TSA-Net PSNR/SSIM	MST PSNR/SSIM	F-MST PSNR/SSIM
Scene 1	30.02/0.896	30.65/0.972	29.08/0.917	37.67/0.983	**38.25**/**0.988**	38.19/0.987
Scene 2	25.95/0.776	27.60/0.883	26.01/0.808	35.10/0.983	35.39/**0.989**	**36.21**/**0.989**
Scene 3	22.07/0.709	20.75/0.687	25.58/0.834	34.98/0.975	35.35/**0.980**	**35.48**/**0.980**
Scene 4	29.91/0.848	27.17/0.833	30.36/0.923	38.34/0.984	38.63/**0.992**	**39.27**/0.990
Scene 5	28.01/0.802	32.89/0.939	28.09/0.886	37.54/0.986	38.34/**0.991**	**39.16**/**0.991**
Scene 6	30.65/0.825	32.84/0.943	29.00/0.879	39.80/0.984	40.93/**0.992**	**41.20**/**0.992**
Scene 7	23.67/0.799	25.82/0.893	23.53/0.903	32.17/0.976	**33.30**/**0.982**	33.12/0.981
Scene 8	28.42/0.836	26.97/0.813	26.70/0.835	36.51/0.982	36.77/**0.987**	**37.18**/**0.987**
Scene 9	26.14/0.816	28.57/0.868	26.98/0.879	36.17/0.981	36.08/0.982	**36.47**/**0.983**
Scene 10	28.04/0.805	32.20/0.947	26.77/0.853	36.65/0.986	37.11/**0.992**	**37.26**/**0.992**
Average	27.29/0.811	28.55/0.878	27.21/0.872	36.49/0.982	37.02/**0.988**	**37.35**/0.987
Average runtime	149.94 s	175.46 s	26.75 s	0.04 s	**0.03** s	0.04 s
Correlation	0.7802	0.8898	0.8968	0.9898	0.9914	**0.9969**
SAM	0.4633	0.3397	0.3161	0.1115	0.1137	**0.0569**

**Table 2:** Reconstruction PSNRs (dB), SSIMs, average runtimes, correlations, and SAMs of different methods based on three selected scenes using real experimental data.

Methods	GPSR PSNR/SSIM	TwIST PSNR/SSIM	GAP-TV PSNR/SSIM	TSA-Net PSNR/SSIM	MST PSNR/SSIM	F-MST PSNR/SSIM
Scene 30	19.98/0.767	22.23/0.807	16.94/0.487	26.91/0.850	26.90/0.865	**27.21**/**0.869**
Scene 31	17.59/0.690	19.79/0.754	22.50/0.762	24.20/0.846	27.53/0.915	**28.78**/**0.918**
Scene 32	22.78/0.845	22.92/0.853	17.63/0.581	29.68/0.890	29.34/**0.906**	**30.69**/0.901
Average	20.12/0.767	21.65/0.805	19.02/0.610	26.93/0.862	27.92/0.895	**28.89**/**0.896**
Average runtime	139.86 s	163.91 s	25.06 s	0.04 s	**0.03** s	0.04 s
Correlation	0.7799	0.7375	0.7724	0.9964	0.9971	**0.9975**
SAM	0.2193	0.2245	0.2534	0.0324	0.0271	**0.0265**

## Data Availability

The datasets presented in this article are not readily available because the data are part of an ongoing study. Requests to access the datasets should be directed to maxu@bit.edu.cn.

## Supplementary Materials

The following supporting information can be downloaded at https://www.mdpi.com/article/10.3390/s25072006/s1. Table S1: Reconstruction PSNRs (dB), SSIMs, correlations and SAMs of different methods based on Scene 8 using simulation data; Table S2: Reconstruction PSNRs (dB), SSIMs, correlations and SAMs of different methods based on Scene 32 using real experimental data; Table S3: Reconstruction MSEs of spectral curves obtained by different methods using the real experimental data of Scene 32; Figure S1: Original and reconstructed results of Scene 8 obtained by different algorithms using simulation data; Figure S2: Original and reconstructed results of Scene 32 obtained by different algorithms using simulation data; Figure S3: The ground truth and reconstructed spectral curves obtained by different methods corresponding to three spatial positions of Scene32.
