# Supplementary material for: Color-Coded Compressive Spectral Imager Based on Focus Transformer Network
Source: Sensors (Basel). 2025 Mar 23;25(7):2006. doi: 10.3390/s25072006 (PMC11990993; doi:10.3390/s25072006)
Supplement: Supplementary file 1 [file sensors-25-02006-s001.zip › sensors-3351140-supplementary.pdf]

Color-Coded Compressive Spectral Imager Based on Focus Transformer Network

Jinshan Li [1], Xu Ma [1,*], Aanish Paruchuri [2], Abdullah Alrushud [2] and Gonzalo R. Arce [2,*]

[1] Key Laboratory of Photoelectronic Imaging Technology and System of Ministry of Education of China, School of Optics and Photonics, Beijing Institute of Technology, Beijing 100081, China; lijinshan0527@163.com

[2] Department of Electrical and Computer Engineering, University of Delaware, Newark, DE 19716, USA; aanishp@udel.edu (A.P.); alrushud@udel.edu (A.A.)

* Correspondence: maxu@bit.edu.cn (X.M.); arce@udel.edu (G.R.A.)

Academic Editors: Barry K. Lavine and Walter Neu

Received: 19 November 2024
Revised: 20 March 2025
Accepted: 21 March 2025
Published: 23 March 2025

Citation: Li, J.; Ma, X.; Paruchuri, A.; Alrushud, A.; Arce, G.R. Color-Coded Compressive Spectral Imager Based on Focus Transformer Network. *Sensors* **2025**, *25*, x. https://doi.org/10.3390/xxxxx

Copyright: © 2025 by the authors. Submitted for possible open access publication under the terms and conditions of the Creative Commons Attribution (CC BY) license (https://creativecommons.org/licenses/by/4.0/).

1. Simulation results of Scene 8

In order to provide readers with a more detailed understanding of the superiority of the algorithm proposed in this article, this section presents the reconstruction results of another simulation data based on six algorithms. Table S1 shows the reconstruction PSNRs and SSIMs of Scene 8 based on different methods. Figures S1(a) and S1(b) show the RGB image and compressive measurement of Scene 8, respectively. The ground truth and the reconstructed spectral curves that correspond to the spatial point marked with a green cross in the RGB image are presented in Fig. S1(c). And the correlations and SAMs of the ground truth and the reconstructed spectral curves are shown in Table S1. In addition, the reconstructed hyperspectral images (HSIs) within four selected spectral bands of six methods are shown in Fig. S1(d). We can observe that F-MST algorithm achieves the highest reconstruction accuracy in all evaluation metrics, further demonstrating its advantages compared to other algorithms.

Table S1. Reconstruction PSNRs (dB), SSIMs, correlations and SAMs of different methods based on Scene 8 using simulation data.

Methods	GPSR	TwIST	GAP-TV	TSA-Net	MST	F-MST
PSNR	28.42	26.97	26.70	36.51	36.77	**37.18**
SSIM	0.836	0.813	0.835	0.982	**0.987**	**0.987**
Correlation	0.9299	0.9190	0.8923	0.9938	0.9963	**0.9979**
SAM	0.2457	0.2663	0.3067	0.0709	0.0545	**0.0428**

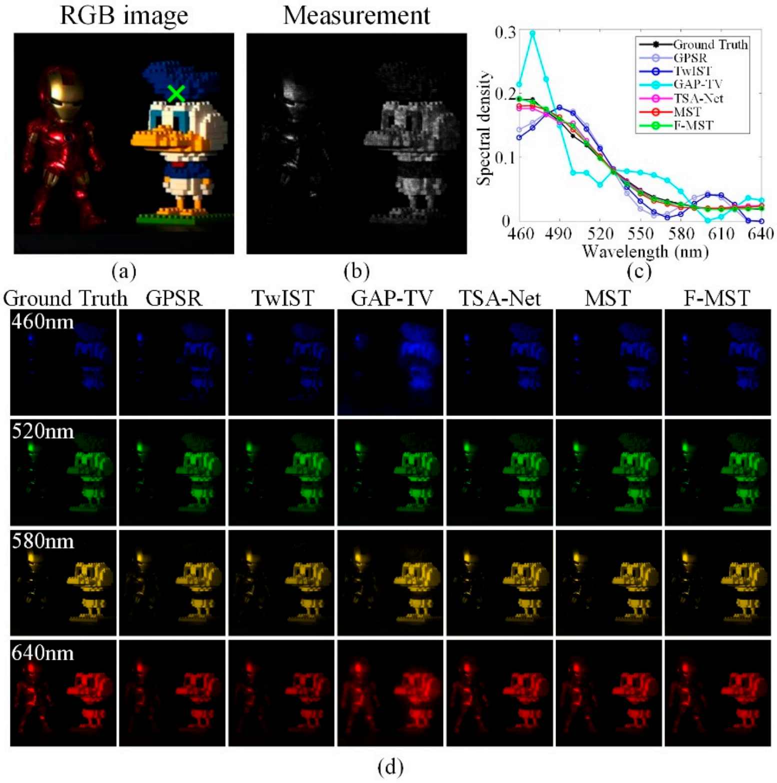

Figure S1. Original and reconstructed results of Scene 8 obtained by different algorithms using simulation data: (a) the RGB image; (b) the compressive measurement; (c) the ground truth and reconstructed spectral curves corresponding to the green cross in the RGB image; (d) the ground truth and reconstructed HSIs within four selected spectral bands.

2. Experimental results of Scene 32

This section presents the experimental results of Scene 32. The reconstruction PSNRs and SSIMs of Scene 32 based on six methods are shown in the first and second rows of Table S2, respectively. Figures S2(a) and S2(b) show the RGB image and compressive measurement of Scene 32, respectively. Figure S2(c) shows the ground truth and reconstructed spectral curves at the location marked by the green cross in RGB image, and the corresponding correlations and SAMs are shown in the last two rows of Table S2. Figure S2(d) shows the ground truth and reconstructed HSIs. Although the reconstruction result of F-MST is slightly inferior to MST algorithm in terms of SSIM, F-MST algorithm shows superiority in comprehensive reconstruction performance compared to other algorithms based on all metrics.

Table S2. Reconstruction PSNRs (dB), SSIMs, correlations and SAMs of different methods based on Scene 32 using real experimental data.

Methods	GPSR	TwIST	GAP-TV	TSA-Net	MST	F-MST
PSNR	22.78	22.92	17.63	29.68	29.34	**30.69**
SSIM	0.845	0.853	0.581	0.890	**0.906**	0.901
Correlation	0.9085	0.9171	0.9527	0.9859	0.9934	**0.9967**
SAM	0.3006	0.2749	0.2358	0.1916	0.1249	**0.0706**

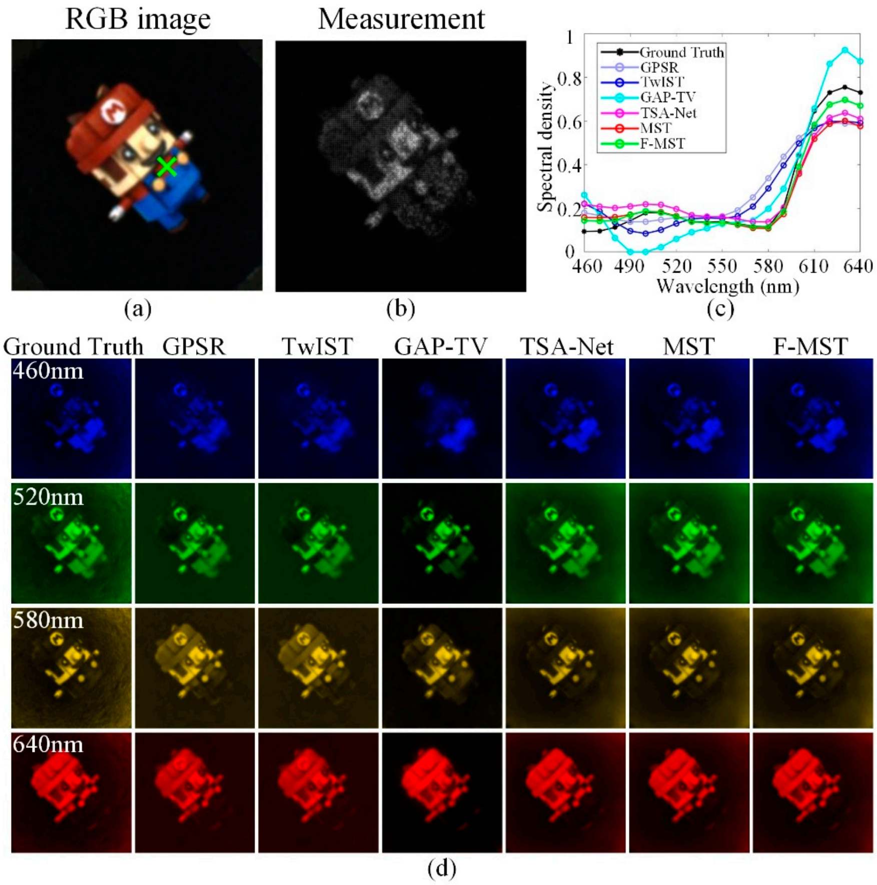

Figure S2. Original and reconstructed results of Scene 32 obtained by different algorithms using simulation data: (a) the RGB image; (b) the compressive measurement; (c) the ground truth and reconstructed spectral curves corresponding to the green cross in the RGB image; (d) the ground truth and reconstructed HSIs within four selected spectral bands.

In order to further demonstrate the advantages of the F-MST network, we present the reconstructed spectral curves corresponding to three additional spatial positions (P1-P3) of Scene 32 in Fig. S3. These spatial positions are marked by the green crosses in the RGB image. Furthermore, we calculate the mean squared errors (MSEs) between the ground truth and the reconstructed spectral curves to assess the reconstruction accuracy of different algorithms, as shown in Table S3. We can observe that the proposed F-MST algorithm significantly outperforms the other five algorithms in terms of the MSE metric. This result apparently demonstrates the advantages of the proposed focus-based down-sampling and up-sampling modules.

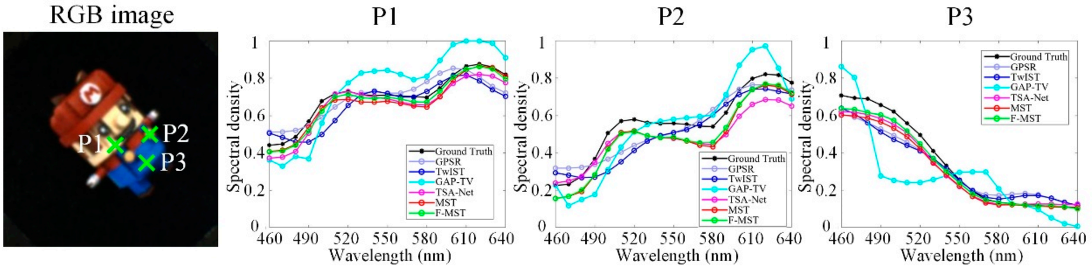

Figure S3. The ground truth and reconstructed spectral curves obtained by different methods corresponding to three spatial positions of Scene32.

Table S3. Reconstruction MSEs of spectral curves obtained by different methods using the real experimental data of Scene 32.

Methods	GPSR	TwIST	GAP-TV	TSA-Net	MST	F-MST
P1	0.0035	0.0062	0.0146	0.0021	0.0012	**0.0008**
P2	0.0068	0.0095	0.0116	0.0084	0.0061	**0.0053**
P3	0.0054	0.0055	0.0307	0.0024	0.0036	**0.0014**
